# Optimization of germination and ultrasonic‐assisted extraction for the enhancement of γ‐aminobutyric acid in pumpkin seed

**DOI:** 10.1002/fsn3.2826

**Published:** 2022-03-21

**Authors:** Li Liang, Lin Chen, Guimei Liu, Fuming Zhang, Robert J. Linhardt, Baoguo Sun, Quanhong Li, Yuyu Zhang

**Affiliations:** ^1^ Beijing Key Laboratory of Flavor Chemistry Beijing Technology and Business University (BTBU) Beijing China; ^2^ 34752 National Engineering Research Center for fruit and vegetable Processing College of Food Science and Nutritional Engineering China Agricultural University Beijing China; ^3^ 12689 School of Food Sciences and Engineering Qilu University of Technology Jinan China; ^4^ 8024 Departments of Chemical and Biological Engineering, Chemistry and Chemical Biology Biomedical Engineering and Biological Science Center for Biotechnology and Interdisciplinary Studies Rensselaer Polytechnic Institute Troy New York USA

**Keywords:** germination, hypolipidemic, pumpkin seeds, ultrasonic‐assisted extraction, γ‐aminobutyric acid

## Abstract

Germination and ultrasonic‐assisted extraction (UAE) are economical and effective methods to enhance bioactive compounds in plant seeds. We optimized the germination parameters and UAE parameters by using response surface methodology to maximize the recovery of γ**‐**aminobutyric acid (GABA) in pumpkin seeds. The optimal germination conditions were as follows: soaking the seeds at 28°C for 6 h with 0.2% CaCl_2_, 3.8 mg/ml monosodium glutamate, and 4.0 mg/ml vitamin B_6_, then germination at 30°C for 61.6 h. The optimal conditions for UAE were as follows: 1:75 (w/v) material‐to‐solvent ratio, 220 W ultrasonic power, and ultrasonic treatment at 50°C for 14.4 min, which afforded an extraction yield of 2679 ± 10 mg/100 g. Moreover, the GABA‐enhanced extract showed the potential of hypolipidemic effect in type 2 diabetes rats. These results confirmed that a combination of germination and UAE increased the GABA yield from pumpkin seeds and provided a basis for GABA‐enhanced production to improve lifestyle‐associated diseases.

## Graphical abstract

By using response surface methodology, the germination and UAE parameters were optimized to enhance GABA in pumpkin seeds: the seeds were soaked at 28°C for 6 h with 0.2% CaCl_2_, 3.8 mg/ml MSG, and 4.0 mg/ml vitamin B_6_, then germinated at 30°C for 61.6 h; UAE were carried out at ratio of 1:75 (w/v), 220 W, and 50°C for 14.4 min. Moreover, the GABA‐enhanced extract showed the potential of hypolipidemic effect in T2DM rats. A combination of germination and UAE increased the GABA recovery from pumpkin seeds. The biochemical parameters of serum in HFD‐fed T2DM rats showed the regulation of TC, TG, and HDL after being treated with GABA‐enhanced extract from germinated pumpkin seeds.

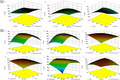

## 1 INTRODUCTION

Pumpkin is a commercially important crop and cultivated worldwide. Pumpkin seeds are easily available and are rich sources of several functional components (Dotto & Chacha, 2020) including unsaturated oils, proteins, phytosterols, vitamin K, and γ**‐**aminobutyric acid (GABA), which has beneficial effects on some lifestyle‐associated diseases. The hypoglycemic and hypolipidemic effects of whole pumpkin seeds have been previously reported (Adams et al., 2011), promoting the acceptance of pumpkin seeds in the individuals with related lifestyle‐associated diseases. Because of the nutritional and potential therapeutic values, interest in the application of pumpkin seeds has considerably increased in recent years. GABA (C_4_H_9_NO_2_), a bioactive constituent of pumpkin seeds, is a 4‐carbon non‐protein amino acid that plays a key role in living organisms (Bown & Shelp, 2016). GABA has many reported bioactivities, such as alleviation of anxiety, regulation of blood pressure and cholesterol, and prevention of obesity and diabetes (Chua et al., 2019; Imam et al., 2012; Sato et al., 2021; Shelp et al., 1999; Wu et al., 2021). Therefore, investigation of GABA‐enriched functional food is becoming increasingly important.

In plant, GABA is synthesized from the l‐glutamic acid (Glu) in plants through catalytic activity of glutamate decarboxylase (GAD) (Bouché & Fromm, 2004). Thus, the concentration of Glu, the activity of GAD, and environmental factors contribute to the accumulation of GABA. Usually, naturally occurring GABA in plants is insufficient to meet the nutritional requirement as a functional food and chemically synthesized GABA is not permitted as a food additive (Wu & Shah, 2017). Accordingly, biochemical methods, such as fermentation, enzymatic hydrolysis, germination, and ultrasonic extraction, have been applied to enrich GABA in natural food production (Ding et al., 2016, 2018; Galli et al., 2022). Several literatures have demonstrated that germination is an economical and effective method for enriching bioactive compounds in plant seeds (Ding et al., 2016; Moongngarm & Saetung, 2010; Ohanenye et al., 2020). During the germination process, biosynthesis and interconversion reactions can result in the generation of new compounds and accumulation of metabolites (Kumar & Chauhan, 1993). Related studies showed that the germination treatment has a profound impact on the accumulation of functional components including GABA (Bhinder et al., 2022; Cáceres et al., 2014). Furthermore, temperature, pH, and other environmental factors can also impact the efficiency of GABA accumulation by altering the extent of germination (Cáceres et al., 2014; Zhang et al., 2014). Several studies have improved the nutritive value in pumpkin seeds through their germination (Quanhong & Caili, 2005). However, there is still a lack of work on the optimization of parameters for the accumulation of GABA in pumpkin seed. Ultrasonic‐assisted extraction (UAE) is regarded as a green process for the extraction of valuable natural products (Naik et al., 2021). UAE has the advantages of high product yields with low maintenance costs and short processing times. Processes relying on UAE are affected by cavitation, thermal, and mechanical conditions. Bubbles formation and growth enhance the chemical activity in the solution when ultrasound transmits in liquid medium (Kiss et al., 2018), therefore, several probable mechanisms for UAE have been proposed. Based on the mechanical and cavitation effectiveness in UAE, the cell wall is disrupted and particle size is reduced by improving the mass transfer across the cell membrane, and the penetration, swelling, as well as hydration process are enhanced (Hossain et al., 2012; Vinatoru & Nenitzescu, 2001; Wang et al., 2008). UAE has been successfully applied to the extraction of functional compounds from grains and beans (Ding et al., 2018; Eze et al., 2022), but there is lack of studies on the optimization of UAE parameters applied in GABA extraction from pumpkin seed.

In this study, germination and UAE were combined to effectively improve the yield of GABA in pumpkin seeds. Response surface methodology (RSM) was applied to optimize the parameters in both the germination and UAE process. The impact of germination on GABA enrichment in pumpkin seed and the effect of UAE on GABA yield of the extraction were also established. Moreover, a rat model for T2DM was applied to reveal the bioactivity potential of the GABA extract from pumpkin seeds. This study aims to provide an economic, green, and efficient method for the production of GABA‐enriched functional food and reveal the potential of pumpkin seeds as a natural nutritional supplement in the daily diet.

## 2 MATERIALS AND METHODS

### 2.1 Materials and reagents

Seven commercial cultivars of seed‐rich pumpkin in China, including Dali, Luoren No.2, Luoren No.3, Jinhui No. 4, Ruifeng No.9, Yinhui No. 3, and Xuecheng No.2 (coded LR‐1, LR‐2, LR‐3, JH‐4, RF‐9, YH‐3, and XC‐2), were purchased from Mudanjiang Beidahuang Food Co., Ltd. All seeds were received in good shape (without damage) and were stored at 4℃.

Gamma‐aminobutyric acid (GABA) and phenylisothiocyanate (PITC) were purchased from Sigma Chemical Co. Monosodium glutamate (MSG), trimethylamine, n‐hexane, methyl alcohol, acetonitrile of HPLC grade, and other analytical reagents were obtained from Sinopharm Chemical Reagent Co., Ltd.

### 2.2 Procedures of germination of pumpkin seeds and UAE treatment

Pumpkin seeds (50 g, dry weight) were soaked in sodium hypochlorite solution (0.1%) for 10 min and rinsed with distilled water until reaching neutral pH. The pumpkin seeds were then soaked at different temperatures (15, 20, 25, 30, 35, and 40°C) and for gradient times (2, 4, 6, 8, 10, 12, and 14 h). The soaking solutions contained gradient concentrations of MSG (0.5, 1.5, 2.5, 3.5, 4.5, and 5.5 mg/ml), CaCl_2_ (0, 0.2%, 0.4%, 0.6%, 0.8%, and 1.0%), vitamin B_6_ (1, 2, 3, 4, 5, and 6 mg/ml), and at different pH values (4.20, 4.60, 5.00, 5.40, 5.80, and 6.20). The samples were then drained and placed on culture dishes with moist cotton gauze in an incubator (DHP‐9082 Yiheng Science Instruments Co., Ltd.). Germination was performed at different temperatures (15, 20, 25, 30, 35, and 40°C) and for various times (12, 24, 36, 48, 60, and 72 h) in the darkness and sprayed with distilled water every 6 h. After 72 h, sprout length (SL) and germination percentage (GP) were calculated. GP was calculated from the following Equation (1):

(1) $$\text{GP}=\left(\text{germinated pumpkin seeds}/\text{total seeds}\right)\times 100\%$$

Germinated seeds of each cultivar were carefully rinsed with distilled water and dried. After crushing, the samples were defatted with n‐hexane (1:5 w/v) on a shaking table at 55°C for 6 h and repeated twice. The solid phase was dried by hot air at 50°C for 2 h and sieved through a 60 mesh screen to obtain powdered samples, which were stored in darkness at −20°C until further analysis. The protein content was determined by the Kjeldahl method (GB 5009.5‐2010), and the automatic amino acid analyzer 835‐30 (Hitachi High‐Technologies Corporation) was used to obtain the amino acid content before and after germination.

After germination, powdered samples were placed in 50‐ml test tubes with various volumes of distilled water (solid–liquid ratio of 1:20, 1:40, 1:50, 1:60, 1:80, and 1:100). After mixing, samples were placed in an ultrasound device (KQ5200DE Kunshan Ultrasonic Instruments Co., Ltd.). The UAE treatment was performed under various operating power settings (100, 200, 250, 300, 400, and 500 W), times (10, 20, 25, 30, 40, and 50 min), and temperatures (20, 30, 35, 40, 50, and 60°C). After ultrasonic treatment, samples were centrifuged at 9391 × *g* for 10 min, then the supernatant was filtered through a 0.45 μm membrane filter and stored in darkness at 4°C awaiting further analysis. When the optimum parameters of germination and UAE optimum conditions were determined, crude GABA extract from pumpkin seeds was freeze dried.

### 2.3 Experimental design and data analysis for optimization

Based on single‐factor experiments and Plackett–Burman design, RSM was applied for the optimum germinant parameters and ultrasonic extraction parameters for high GABA content. Central composite design (CCD) was used to determine the combined effects of soaking temperature, MSG concentration, and germination time on GABA content. The experimental runs in CCD are shown in Table S1. Similarly, the four independent variables (solid–liquid ratio, ultrasonic power, time, and temperature) for UAE were run in a Box–Behnken design (BBD) to determine the optimum combinations for GABA yield. Detailed information of factors and levels are presented in Table S2.

The variation in GABA yield related to the independent variables was evaluated by a second‐order polynomial regression model, as shown in Equation (2), which is a widely accepted empirical model in optimization process (Myer & Montgomery, 2002). According to Equation (2), linear, quadratic, and interactive effects of independent variables (*X*) on dependent variable (*Y*) were determined.

(2) $$Y=\beta_{0}+\sum \beta_{i}X_{i}+\sum \beta_{\mathrm{ii}}X_{i}^{2}+\sum \beta_{\mathrm{ij}}X_{i}X_{j}$$

where *Y* denotes the response variable obtained from treatment combination of independent variables (*X_i_*, *X_i_*_+1_, *X_j_*), *β*_0_ represents the intercept, and *β_i_*_,_ *β_ii_*_,_ and *β_ij_* are the linear, quadratic, and interaction regression coefficients of variables, respectively.

### 2.4 GABA measurement

The content of GABA was calculated by using HPLC with precolumn PITC derivatization using a modified previously described method (Qingyun et al., 2008). Defatted sample (0.1 g) was mulled with 5 ml of distilled water for ultrasonic extraction, which lasted 20 min at 37℃, and then centrifuged. A 400 μl aliquot of the supernatant or standard GABA solution (0.50, 0.30, 0.20, 0.15, 0.10, 0.08, and 0.05 mg/ml) was mixed with 200 µl of 0.1 mol/L PITC‐acetonitrile solution and 400 µl of 1 mol/L trimethylamine‐acetonitrile solution. After holding for 1 h in darkness, 1.0 ml of n‐hexane was added, and the mixture was shaken and on standing separated. The lower phase was collected and filtered through a 0.45 μm membrane filter. HPLC (LC‐20A Shimadzu Co. Ltd) was applied to quantify GABA content. Samples (10 μl) were loaded onto a Venusil AA column (250 × 4.6 mm, 5 μm) with a temperature of 30°C and detected at 254 nm. The chromatogram was developed at a flow rate of 0.9 ml/min by eluting the sample in mobile phase A (0.02 mol/L sodium acetate buffer solution treated with 200 μl of triethylamine per liter of buffer, pH 6.0) and mobile phase B (acetonitrile), and the elution gradient is listed in Table S3.

### 2.5 Animal experiments

Animal experiment was carried out referring to the reported method (Liu et al., 2018) with some modification. Besides, all the experimental protocol was approved by the Ethics Committee of Beijing Laboratory Animal Research Center (SYXK2015‐0046; Beijing, China). Adult male Wistar rats were obtained and kept in cages with SPF barrier environment (temperature of 21–23°C, relative humidity of 40%–45%, and 12 h light/dark cycle). All the rats had free access to food and water for 1 week in order to acclimatize. The rats received high‐fat diet (HFD) (10% lard, 20% sucrose, 2.5% cholesterol, 1% sodium cholate, and 66.5% pulverized standard rat pellet) for 4 weeks for adaptation with the diet, and then the rats were injected with streptozocin (STZ) (30 mg/kg body weight) after adaptations. The presence of polyuria and polydipsia along with fasting blood glucose higher than 11.1 mol/L indicated that the T2DM rat model was established successfully. The T2DM rats were randomly separated into three groups of 10 rats each: (1) control group (T2DM group), continually fed with HFD diet and intragastrically administered with saline; (2) Metformin (MT) treatment group (MT group), fed with HFD diet and intragastrically administered with 200 mg/kg body weight metformin once daily; (3) GABA‐enhanced extract from germinated pumpkin seeds (PSGE) treatment group (PSGE group), fed with HFD diet and intragastrically administered with 2 g/kg body weight of PSGE (GABA content is 25.0 ± 0.8 mg/g) once daily (Tian et al., 2011).

At the end of the experiment, blood samples were collected and promptly centrifuged to isolate the serum. Then, the serum was kept at a temperature of −60°C for biochemical analysis, including blood glucose (BG), total cholesterol (TC), triglyceride (TG), high‐density lipoprotein cholesterol (HDL‐c), low‐density lipoprotein cholesterol (LDL), and other blood biochemical parameters (Zhang et al., 2017).

## 3 RESULTS AND DISCUSSION

### 3.1 Selection of pumpkin seeds varieties for GABA accumulation

**FIGURE 1:**
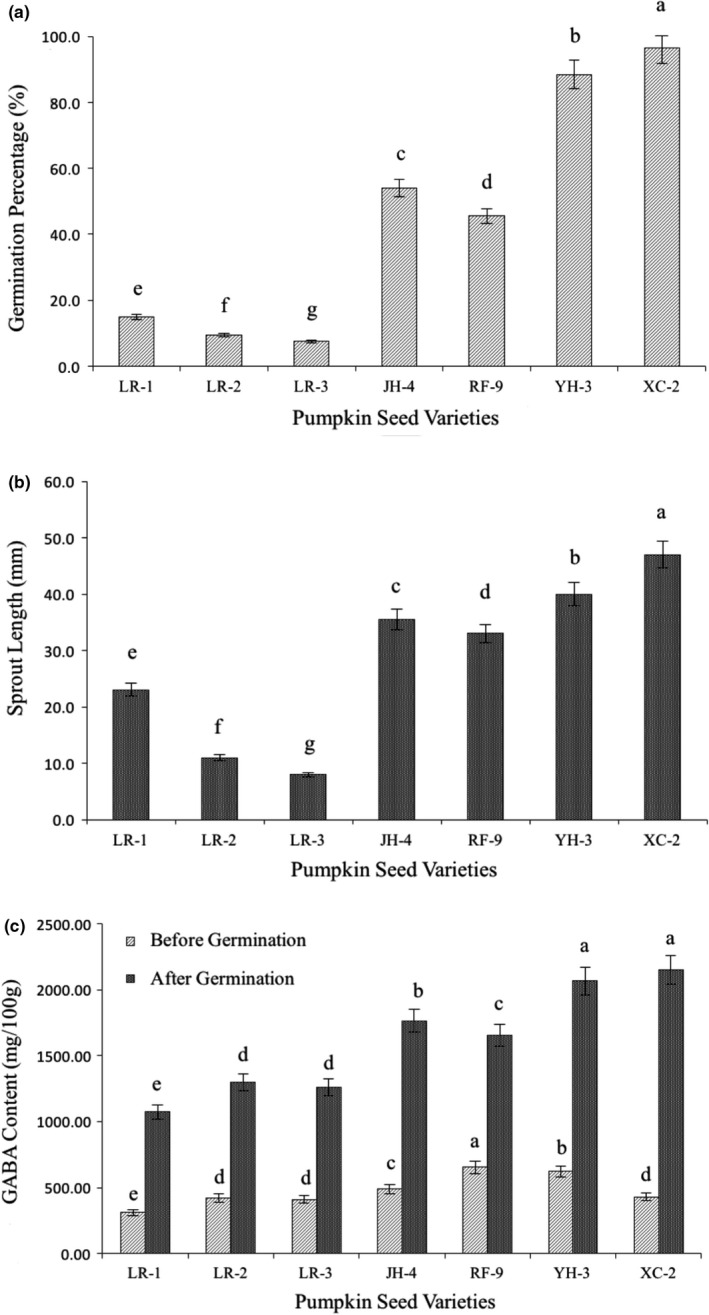
Variation in germination percentage, sprout length, and GABA content in pumpkin seeds before and after 72 h germination

In present study, sprout length (SL), germination percentage (GP), GABA content, protein, and amino acid content were evaluated among seven cultivars after germinating for 72 h. The germination percentage and sprout length reflect the growth status of pumpkin seeds. As shown in Figure 1a,b, significant differences were observed among cultivars. YH‐3 and XC‐2 germinated effectively under the experimental condition, which resulted in germination percentage and sprout length of 89%, 40.0 mm and 97%, 47.0 mm, respectively. Figure 1c shows the changes in GABA content. For all the tested cultivars, the GABA content was significantly increased (*p* < .01) after germination. Before germination, RF‐9 and YH‐3 exhibited higher GABA content (653 mg/100 g, 622 mg/100 g), while the GABA content of other cultivars range from 310 mg/100 g to 488 mg/100 g. After germination for 72 h, GABA accumulated in all cultivars, but maximum GABA content (2150 mg/100 g) was found in XC‐2. This was an increase of five times present in raw seeds. Based on GABA content after germination, the sequence was XC‐2 > YH‐3 > JH‐4 > RF‐9 > LR‐2 > LR‐3 > LR‐1. It has been reported that soybeans with lower initial GABA content show higher GABA accumulation efficiency after germination (Huang et al., 2017). This is in agreement with our current study on the different pumpkin seeds cultivars.

Changes in total protein (TP) and amino acid (AA) content in pumpkin seeds were also affected by germination. The TP content of YH‐3, LR‐3, RF‐9, XC‐2, and LR‐2 diminished after germination (Table 1). During germination, part of the protein in seeds is degraded, providing energy and nutrition for seeds to grow. TP of LR‐1 and JH‐4 increased but these differences were not statistically significant. Meanwhile, AA content of YH‐3, LR‐3, RF‐9, XC‐2, and LR‐2 was increased after 72 h of germination. In addition, the AA content of XC‐2 was significantly higher than other cultivars. The enhancement of essential amino acid contents was observed in a previous study of germinated rice (Kamjijam et al., 2021), which is consistent with the results in this study. Combined with the results of protein content, we conclude that part of macromolecular protein is converted into amino acids during germination (Guo et al., 2011). In addition, the 27% decrease in AA in LR‐1 may be caused by new protein synthesis in the late period of seed germination. Furthermore, during germination, GAD was activated, resulting in the conversion of Glu to GABA (Komatsuzaki et al., 2007). Thus, the change in glutamic acid content plays an important role in the production of GABA. Among the seven tested cultivates, YH‐3 and XC‐2 had the highest content of Glu, which were 20.9% and 20.8%, respectively. After germination, the proportion of Glu in pumpkin seeds decreased slightly. We speculate that Glu is converted into GABA under the action of GAD or participated in some biochemical reactions such as protein synthesis during seed germination.

**TABLE 1:** Changes in protein and amino acid content in pumpkin seeds before and after germination

Varieties	Total protein (g/100 g)		Total amino acids (g/100 g)		Glutamate (g/100 g)	
	Before	After	Before	After	Before	After
LR‐1	53.0 ± 0.7 a	53.2 ± 0.9 a	72.1 ± 1.5 a	52.6 ± 0.9 e	14.8 ± 0.38 a	10.6 ± 0.4 e
LR‐2	48.6 ± 0.9 c	46.0 ± 1.2 d	58.6 ± 0.8 cd	60.5 ± 1.2 c	12.0 ± 0.2 de	12.2 ± 0.1 c
LR‐3	47.9 ± 1.1 c	46.6 ± 0.6 d	49.0 ± 1.6 e	50.2 ± 1.0 f	10.1 ± 0.1 f	10.2 ± 0.4 e
JH‐4	48.5 ± 0.4 c	49.2 ± 0.6 c	57.7 ± 1.1 d	57.0 ± 0.8 d	11.9 ± 0.3 e	11.5 ± 0.2 d
RF‐9	50.4 ± 1.2 b	48.7 ± 0.6 c	60.5 ± 0.8 c	62.5 ± 1.1 b	12.4 ± 0.3 d	12.7 ± 0.3 b
YH‐3	52.0 ± 0.8 a	50.7 ± 0.4 b	61.0 ± 0.8 bc	62.3 ± 0.2 b	12.7 ± 0.1 c	12.8 ± 0.2 b
XC‐2	52.3 ± 0.5 a	50.1 ± 1.0 bc	62.5 ± 0.7 b	65.3 ± 0.5 a	13.0 ± 0.3 b	13.4 ± 0.2 a

Note

Data were presented as mean ± SD (*n* = 3). Values in the same row with different superscript letters (a–f) are significantly different (*p* < .05) in the same group.

Correlation analysis (Table 2) was conducted on parameters. GP was positively associated with sprout length (*r* = .941) and AA (*r* = .739), indicating that cultivar with higher TP is more able to germinate and grow well. Meanwhile, GABA content had obviously positive correlation with GP, SL, TP, and GLU content, and the correlation coefficient are 0.97, 0.87, 0.77, and 0.74, respectively. Therefore, good conditions for growth and nutrition utilization along with large content of AA and Glu could promote the accumulation of GABA during germination. Among all these tested cultivars, XC‐2 had better germination percentage and sprout length after light avoid germination for 72 h. Compared with original pumpkin seeds, the AA and Glu content substantially increased in XC‐2 after germination. Apart from that, the largest content and increment of GABA after germination directly elaborated the effective GABA enrichment in XC‐2, making it suitable for the development of GABA‐enriched food. In conclusion, XC‐2 was considered as a better selection for GABA accumulation in the present study.

**TABLE 2:** Correlation analysis between physiological indexes and main components of pumpkin seeds

	Germination percentage	Sprout length	Total protein	Total amino acids	Glutamate
Sprout length	0.94**				
Total protein	0.36	0.53*			
Total amino acids	0.74**	0.69**	0.042		
Glutamate	0.73**	0.68**	0.016	0.94**	
GABA	0.97**	0.87**	0.16	0.77**	0.74**

* Significantly different (*p* < .05)

** Extremely significantly different (*p* < .01).

### 3.2 Response surface analysis of GABA germination

The results of Plackett–Burman design and significant analysis are shown in Tables S4 and S5. Among all the eight factors, germination temperature, MSG concentration, and germination time had significant effects on GABA content of XC‐2, then we finally determined the optimum parameters of germination conditions by CCD (Table S6). The design model was found to be consistent with the second‐order polynomial model through multiple regression analysis. The relationship between GABA content and germination parameters was shown in the following equation:

$$\begin{array}{c} \begin{array}{cc} \;Y_{1} & \;\;=2122.75‐34.45A_{1}‐74.27B_{1}‐45.87C_{1}+20.43A_{1}B_{1}\\ & +37.75A_{1}C_{1}+70.06B_{1}C_{1}‐22.74A_{1}^{2}+15.42B_{1}^{2}‐62.29C_{1}^{2} \end{array} \end{array}$$

where *Y*_1_ is GABA content (mg/100 g), and *A*_1_, *B*_1_, and *C*_1_ represent soaking temperature, MSG concentration, and germination time, respectively.

The statistical analysis is listed in Table S6. To assess how well a model explains and predicts future outcomes, the coefficient of determination (*R* ^2^) is calculated as the proportion of the variance in the dependent variable that is predictable from the independent variables (Badwaik et al., 2012). Larger *F*‐value and smaller *p*‐value represent a more significant effect on the response variable, besides, if the lack of fit value was found to be not significant (*p* > .05), it implies that the model fits well (Quanhong & Caili, 2005). $R_{1}^{2}$ value of 0.88 was obtained, which shows the relationship among the GABA content and independent variables. The model possessed an *F*‐value of 5.72, indicated the model was very significant (*p* = .006 < .01). And the lack of fit value was found to be not significant (*p* = .41 > .05), which proved the validity of the experimental model. The analysis of variance (ANOVA) showed that GABA content was significantly (*p* < .05) affected by MSG concentration and germination time, being the effect of germination time higher than MSG concentration, and the result was consistent with the germination study on rice (Kamjijam et al., 2021). Additionally, germination time and MSG concentration interacted significantly on GABA accumulation during germination.

**FIGURE 2:**
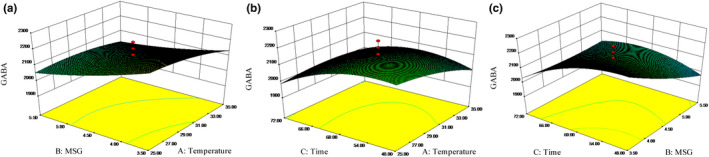
Response surface plots of germination parameters

Response surfaces plots (Figure 2) illustrated the interaction of germination temperature, MSG concentration, and germination time on GABA accumulation. The effects of germination temperature and MSG concentration during germination are presented in Figure 2a. MSG concentration exhibited significant linear effects (*p* < .05). When germination temperature was constant, MSG addition resulted in gradual decline in GABA content. However, the result did not show a significant (*p* > .05) interaction between the two independent variables (Table S6). Supplementation with glutamate can improve GABA accumulation, nevertheless, the decline in GABA content in grain with increasing glutamate concentration was reported owing to the increased enzyme–substrate complex concentration and decreasing GABA conversion rate (Iimure et al., 2009). The effects of germination temperature and germination time on GABA content are revealed in Figure 2b. Germination time had a very significant linear and quadratic effects (*p* < .01) on GABA accumulation. When germination temperature was set, the GABA content increased with germination time and declined later. But, the two independent variables did not interact significantly (*p* > .05) during the experiment. Soaking in water stimulates the enzymes activity and GABA accumulation, but too long a duration may decrease the GABA content attributed to the growth of seedling (Chungcharoen et al., 2014). The effects of MSG concentration and germination time on GABA content are shown in Figure 2c. The GABA content increased with germination time. The highest point occurred in 61.6 h, then it decreased. And the interaction between MSG concentration and germination time was significant (*p* < .05).

By means of the RSM test results, the optimum germination parameters for GABA accumulation were soaking at 28°C for 6 h, CaCl_2_ concentration of 0.2%, MSG concentration of 3.8 mg/ml, vitamin B6 concentration of 4.0 mg/ml, and germination at 30°C during 61.6 h with pH 5.8. The predicted value of GABA content was 2336 mg/100 g. Under optimized conditions, the model verification was performed. The actual content of GABA was 2319 ± 10 mg/100 g and the relative error was only 0.7% when compared with predicted value. The results above indicated that the model was valid; besides, the combination of Plackett–Burman design and CCD method was reliable. After germination, the GABA content was 5.4 times higher than that of the nongerminated XC‐2.

### 3.3 Response surface analysis of UAE

Based on the result of single‐factor experiments, Box–Behnken design was performed to optimize the parameters of UAE conditions, including solid–liquid ratio, ultrasonic power, time, and temperature.

According to the result of multiple regression analysis, the design model was found to be consistent with the second‐order polynomial model. The relationship between GABA yield and UAE parameters is shown in the following equation:

$$\begin{array}{cc} Y_{2} & =2643.64+122.82A_{2}+14.00B_{2}‐18.33C_{2}+65.18D_{2}+80.20A_{2}B_{2}+89.68A_{2}C_{2}\\ & ‐161.29A_{2}D_{2}+152.58B_{2}C_{2}‐88.32B_{2}D_{2}‐46.79C_{2}D_{2}\\ & ‐427.29A_{2}^{2}‐70.78B_{2}^{2}‐109.16C_{2}^{2}‐41.09D_{2}^{2} \end{array}$$

where *Y*_2_ is GABA yield (mg/100 g), and *A*_2_, *B*_2_, *C*_2_, and *D*_2_ are solid–liquid ratio, ultrasonic power, time, and temperature, respectively.

The statistical analysis is listed in Table S7. The model was significant with a satisfactory value of $R_{2}^{2}$ ($R_{2}^{2}$ = 0.92). An *F*‐value of 12.03 and *p* < .0001 indicated the model was highly significant, besides, the lack of fit value was found to be not significant (*p* = .20), which proved the validity of the model. The ANOVA results showed that solid–liquid ratio has a highly significant (*p* < .01) effect on GABA yield. Meanwhile, GABA yield was significantly affected by ultrasonic time and temperature. Additionally, interaction between solid–liquid ratio and ultrasonic temperature showed highly significant effect on GABA yield, ultrasonic power, and time interacted in the same way.

**FIGURE 3:**
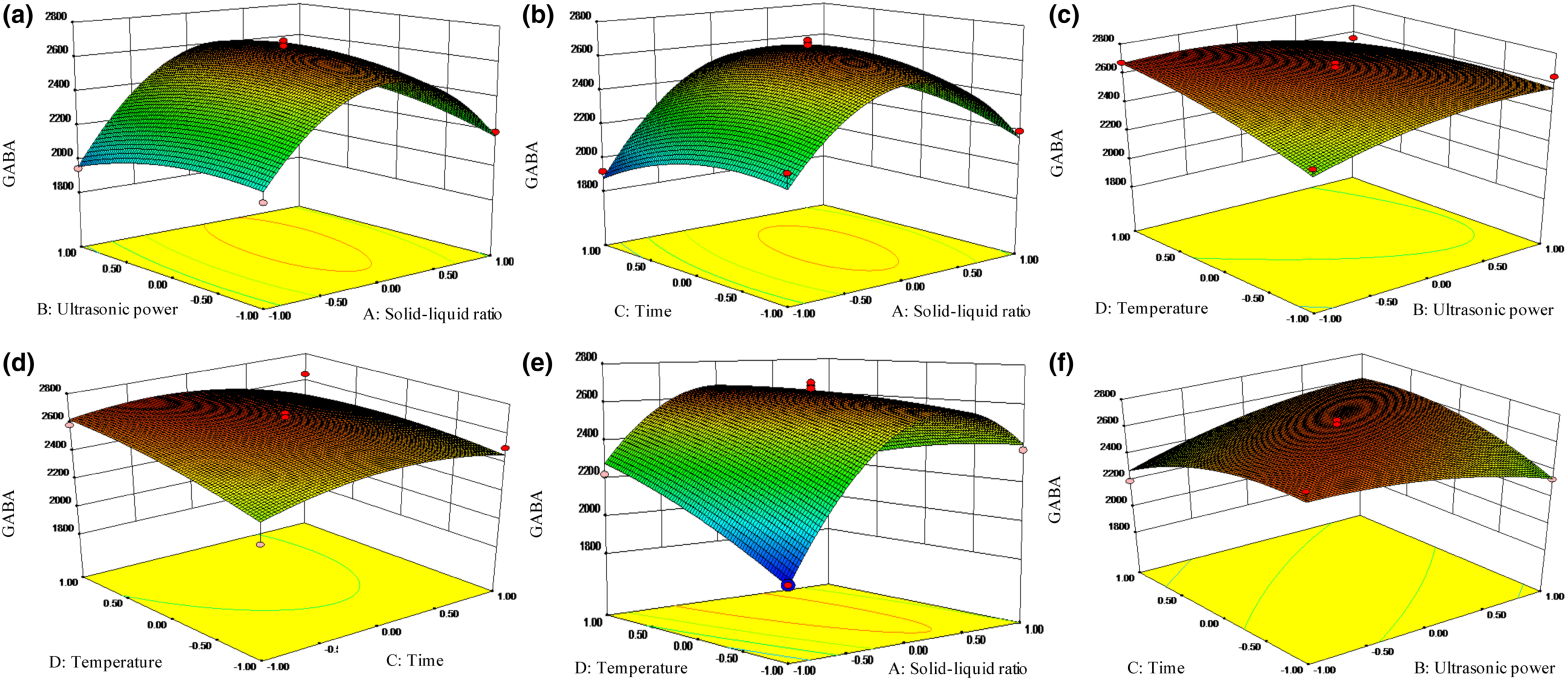
Response surface plots of UAE parameters

Response surfaces plots (Figure 3) were also applied to illustrate the interaction of solid–liquid ratio, ultrasonic power, time, and temperature on GABA extraction efficiency. Effects of solid–liquid ratio and ultrasonic power on GABA extraction are revealed in Figure 3a. Solid–liquid ratio had a very significant (*p* < .01) effect on GABA yield at linear and quadratic level. When ultrasonic power was constant, GABA yield was sharply increased and then descended with the raise in solid–liquid ratio. However, the interaction between solid–liquid ratio and ultrasonic power was not significant (*p* > .05) (Table S7). The effects of solid–liquid ratio and ultrasonic time during UAE treatment are presented in Figure 3b. The two independent variables had quadratic effects on GABA yield during extraction. According to the variation trend, the effect of solid–liquid ratio contributed more to GABA yield. Also, no significant (*p* > .05) interaction was observed between the two independent variables. Figure 3c illustrates the effects of ultrasonic power and temperature during extraction. The two variables had linear effects on GABA yield. With a fixed ultrasonic power, GABA yield rose gradually along with the increase in temperature. Ultrasonic power had a positive effect on GABA yield within a certain temperature range, attributed to high cavitation effect and solvent dispersion effect. The effect of extraction time and temperature on GABA yield is shown in Figure 3d. Ultrasonic time had a significant effect (*p* < .05) at linear level, but the main effect of ultrasonic temperature had a greater impact on GABA yield. The increase in temperature promoted molecular thermal motion, which increased the solubility of GABA. However, the dissolution of other component in pumpkin seeds also increased when the system was overheated (Pinelo et al., 2005). The effect of ultrasonic temperature at linear lever is revealed in Figure 3e, meanwhile, solid–liquid ratio had quadratic effects during UAE treatment. Moreover, solid–liquid ratio and ultrasonic temperature interacted significantly (*p* < .01) on GABA yield. Both ultrasonic power and time had quadratic effects as shown in Figure 3f. The GABA yield was first increased and then decreased along with the increase in the two variables. Additionally, there was a highly significant (*p* < .01) interaction between the two variables. From all the results above, the effect sequence of four variables to GABA yield is as follows: solid–liquid ratio > ultrasonic temperature > ultrasonic time > ultrasonic power.

Considering the practicability, the optimum UAE parameters for GABA extraction were as follows: 1:75 (w/v) material–solvent ratio, 220 W ultrasonic power, and ultrasonic treatment at 50°C for 14.40 min. Under optimized conditions, the model verification was performed. The actual extraction efficiency of GABA was 2679 ± 10 mg/100 g according to the optimized condition; the error with the predicted was only 2.5%. When compared with GABA content in germinated pumpkin seeds, the GABA yield increased by 15.5% under the optimal UAE conditions. The results above indicated that the model was valid.

### 3.4 Hypolipidemic effect of PSGE in T2DM rats

T2DM rat model was established by HFD feeding and injection of STZ. Then, two groups of T2DM rats were treated with PSGE and MT, respectively. After 4 weeks, biochemical parameters of serum were determined and the results are listed in Table 3. The level of TC, TG, and LDL was significantly (*p* < .05) reduced after treating with PSGE as compared with the T2DM group. Moreover, the level of LDL in PSGE group was significantly lower than that in the MT group. The results above were consistent with a study in which the regulation effects of GABA on lipid levels in serum were reported (Rashmi et al., 2018; Sato et al., 2021). Besides, it is demonstrated that germinated brown rice could reduce the level of TC, TG, and LDL (Hsu et al., 2008; Roohinejad et al., 2010), which may relate to GABA accumulation during germination experiments on animals. Several studies reported that insulin sensitivity and glucose tolerance have been regulated by feedings of GABA by experiments on animals (Tian et al., 2011). However, no significant difference was observed in the level of BG (*p* = .397), HDL (*p* = .223), and INS (*p* = .577) among the three groups. As compared with other studies (Tian et al., 2011), short‐term PSGE treatment in the present study may bring about the absent of significant changes in insulin and BG.

**TABLE 3:** The effects of PSGE on serum biochemical parameters of T2DM rats

Biochemical Parameters	T2DM	MT	PSGE	*p‐*Value
BG (mmol/L)	26.2 ± 9.4	21.3 ± 7.0	20.8 ± 8.1	.40
TG (mmol/L)	1.7 ± 0.6 a	1.5 ± 1.1 ab	0.6 ± 0.2 bc	<.05
TC (mmol/L)	5.1 ± 2.4 a	3.4 ± 1.2 ab	2.3 ± 0.5 bc	<.05
LDL (mmol/L)	1.8 ± 1.0 a	1.7 ± 0.6 a	0.7 ± 0.2 b	<.05
HDL (mmol/L)	1.1 ± 0.2	1.2 ± 0.2	1.0 ± 0.2	.22
INS (μIU/ml)	44 ± 11	49.7 ± 11.7	48.8 ± 5.8	.58

Note

Data are presented as mean ± SD. Values in the same row with different letters (a–c) are significantly different, *p* < .05.

Hyperglycemia and dyslipidemia are the typical symptoms of diabetes mellitus. It is caused by a group of metabolic disorders over a long period of time, and the abnormal levels of TC, TG, and LDL lead to the macrovascular complication of T2DM. Due to the side effects of drugs, there is a tendency to use natural ingredients with antidiabetic effects as a supplement for the prevention and treatment of T2DM. In this study, PSGE showed potential hypolipidemic effect on T2DM rats. The extracts from germinated pumpkin seeds may be a good dietary supplement for T2DM patients and satisfy the demand for GABA‐enriched health beneficial foods.

## 4 CONCLUSION

The combined technique of germination and UAE was effective for the enhancement of GABA yield in pumpkin seed. The germination parameters and UAE parameters were successfully screened by BBD and CCD of RSM. The optimal germinated conditions were as follows: soaking the seeds at 28°C for 6 h with 0.2% CaCl_2_, 3.8 mg/ml monosodium glutamate, and 4.0 mg/ml vitamin B_6_, then germination was carried out at 30°C for 61.6 h. After germination with the optimal conditions, the GABA content of XC‐2 was 5.40 times higher than that of raw material. The optimal UAE conditions were as follows: 1:75 (w/v) material‐to‐solvent ratio, 220 W ultrasonic power, and ultrasonic treatment at 50°C for 14.4 min. The GABA yields further increased by 15.5% under the optimal UAE treatment. Furthermore, the regulation of TC, TG, and HDL was observed in T2DM rats after being treated with PSGE, which demonstrated the potential hypolipidemic effect of GABA extract from the pumpkin seed. In conclusion, the application of germination and UAE efficiently provided a GABA‐enriched production of pumpkin seed, which could contribute to health benefits related to lifestyle‐associated diseases.

## CONFLICT OF INTEREST

The authors have declared no conflict of interest.

## AUTHOR CONTRIBUTIONS

**Li Liang:** Formal analysis (equal); Investigation (equal); Visualization (equal); Writing – original draft (equal). **Lin Chen:** Data curation (equal); Methodology (equal); Software (equal). **Guimei Liu:** Investigation (equal); Methodology (equal). **Fuming Zhang:** Formal analysis (equal); Validation (equal); Writing – review & editing (equal). **Robert J. Linhardt:** Writing – review & editing (equal). **Baoguo Sun:** Resources (equal); Supervision (equal). **Quanhong Li:** Conceptualization (equal); Supervision (equal); Writing – review & editing (equal). **Yuyu Zhang:** Resources (equal); Supervision (equal); Writing – review & editing (equal).

## Supporting information

Supplementary Material
