# Supplementary material for: Optimization of germination and ultrasonic‐assisted extraction for the enhancement of γ‐aminobutyric acid in pumpkin seed
Source: Food Sci Nutr. 2022 Mar 21;10(6):2101–10. doi: 10.1002/fsn3.2826 (PMC9179130; doi:10.1002/fsn3.2826)
Supplement: Supplementary file 1 — Supplementary Material [file FSN3-10-2101-s001.doc]

Table S1 Test and result of CCD

| Run | A_1_: Soaking Temperature (℃) | B_1_: MSG Concentration (mg/mL) | C_1_: Germination Time (h) | Response_1_: GABA (mg/100g) |
| --- | --- | --- | --- | --- |
| 1 | 25.00 | 5.50 | 48.00 | 2023.56±45.24 |
| 2 | 21.59 | 4.50 | 60.00 | 2080.23±18.19 |
| 3 | 30.00 | 4.50 | 39.82 | 1966.47±30.66 |
| 4 | 30.00 | 4.50 | 60.00 | 2216.84±34.10 |
| 5 | 35.00 | 5.50 | 48.00 | 1975.53±43.40 |
| 6 | 25.00 | 5.50 | 72.00 | 2034.19±46.08 |
| 7 | 30.00 | 6.18 | 60.00 | 1987.22±38.29 |
| 8 | 30.00 | 4.50 | 60.00 | 2177.32±45.67 |
| 9 | 25.00 | 3.50 | 48.00 | 2393.35±17.94 |
| 10 | 30.00 | 4.50 | 60.00 | 2088.87±40.01 |
| 11 | 30.00 | 4.50 | 60.00 | 2137.89±41.46 |
| 12 | 30.00 | 4.50 | 60.00 | 2068.31±30.39 |
| 13 | 30.00 | 4.50 | 60.00 | 2052.44±22.65 |
| 14 | 35.00 | 5.50 | 72.00 | 1990.62±53.95 |
| 15 | 30.00 | 4.50 | 80.18 | 1896.65±47.65 |
| 16 | 35.00 | 3.50 | 48.00 | 2117.06±34.45 |
| 17 | 30.00 | 2.82 | 60.00 | 2315.53±45.72 |
| 18 | 38.41 | 4.50 | 60.00 | 2006.61±40.51 |
| 19 | 25.00 | 3.50 | 72.00 | 1977.22±69.04 |
| 20 | 35.00 | 3.50 | 72.00 | 1998.45±34.99 |

Table S2 Test and result of BBD

| Run | A_2_: Solid-liquid Ratio (g/mL) | B_2_: Ultrasonic Power (W) | C_2_: Ultrasonic Time (min) | D_2_: Ultrasonic Temperature (℃) | Response2: GABA (mg/100 g) |
| --- | --- | --- | --- | --- | --- |
| 1 | 1:100 | 200 | 20 | 40 | 2197.23 |
| 2 | 1:60 | 200 | 20 | 40 | 2031.57 |
| 3 | 1:80 | 300 | 10 | 40 | 2336.08 |
| 4 | 1:60 | 300 | 20 | 40 | 1942.83 |
| 5 | 1:100 | 250 | 30 | 40 | 2296.14 |
| 6 | 1:80 | 200 | 20 | 30 | 2408.34 |
| 7 | 1:80 | 250 | 20 | 40 | 2689.46 |
| 8 | 1:100 | 250 | 10 | 40 | 2196.12 |
| 9 | 1:100 | 300 | 20 | 40 | 2429.30 |
| 10 | 1:60 | 250 | 30 | 40 | 1917.76 |
| 11 | 1:60 | 250 | 20 | 50 | 2219.67 |
| 12 | 1:80 | 200 | 20 | 50 | 2674.20 |
| 13 | 1:80 | 200 | 10 | 40 | 2688.78 |
| 14 | 1:100 | 250 | 20 | 50 | 2108.92 |
| 15 | 1:80 | 300 | 20 | 50 | 2557.43 |
| 16 | 1:80 | 250 | 20 | 40 | 2616.74 |
| 17 | 1:80 | 250 | 10 | 50 | 2585.20 |
| 18 | 1:80 | 300 | 30 | 40 | 2455.63 |
| 19 | 1:80 | 250 | 20 | 40 | 2531.51 |
| 20 | 1:80 | 200 | 30 | 40 | 2198.02 |
| 21 | 1:80 | 250 | 30 | 50 | 2646.55 |
| 22 | 1:100 | 250 | 20 | 30 | 2365.28 |
| 23 | 1:80 | 250 | 20 | 40 | 2675.16 |
| 24 | 1:60 | 250 | 20 | 30 | 1830.89 |
| 25 | 1:60 | 250 | 10 | 40 | 2176.44 |
| 26 | 1:80 | 300 | 20 | 30 | 2644.86 |
| 27 | 1:81 | 250 | 20 | 40 | 2705.35 |
| 28 | 1:82 | 250 | 10 | 30 | 2255.96 |
| 29 | 1:83 | 250 | 30 | 30 | 2504.49 |

Table S3 Elution procedure for GABA measurement

| Time (min) | Mobile phase A (%) | Mobile phase B (%) |
| --- | --- | --- |
| 0  0.01  3  5  9  13  17  20  22  22.01 | 100  95  90  85  15  10  5  0  95  100 | 0  5  10  15  85  90  95  100  5  0 |

Table S4 Test and results of Plackett-Burman design

| Run | A:  Soakig temperature (℃) | B:  Soaking time (h) | C:  CaCl_2_ (%) | D:  MSG (mg/mL) | E:  VB_6_ (mg/mL) | F:  pH | G:  Germination  temperature (℃) | H:  Germination  time (h) | Response  GABA (mg/100 g) |
| --- | --- | --- | --- | --- | --- | --- | --- | --- | --- |
| 1 | 25.00 | 8.00 | 0.00 | 5.50 | 5.00 | 5.40 | 35.00 | 72.00 | 2217.84 |
| 2 | 25.00 | 4.00 | 0.40 | 3.50 | 5.00 | 6.20 | 25.00 | 72.00 | 2036.47 |
| 3 | 25.00 | 8.00 | 0.40 | 3.50 | 5.00 | 6.20 | 35.00 | 48.00 | 1943.25 |
| 4 | 25.00 | 4.00 | 0.00 | 5.50 | 3.00 | 6.20 | 35.00 | 48.00 | 2032.44 |
| 5 | 35.00 | 4.00 | 0.40 | 5.50 | 5.00 | 5.40 | 25.00 | 48.00 | 2186.90 |
| 6 | 35.00 | 4.00 | 0.40 | 5.50 | 3.00 | 6.20 | 35.00 | 72.00 | 2375.53 |
| 7 | 35.00 | 8.00 | 0.40 | 3.50 | 3.00 | 5.40 | 35.00 | 48.00 | 2007.23 |
| 8 | 35.00 | 8.00 | 0.00 | 5.50 | 5.00 | 6.20 | 25.00 | 48.00 | 2115.42 |
| 9 | 35.00 | 4.00 | 0.00 | 3.50 | 5.00 | 5.40 | 35.00 | 72.00 | 2276.35 |
| 10 | 25.00 | 8.00 | 0.40 | 5.50 | 3.00 | 5.40 | 25.00 | 72.00 | 2352.78 |
| 11 | 25.00 | 4.00 | 0.00 | 3.50 | 3.00 | 5.40 | 25.00 | 48.00 | 1950.36 |
| 12 | 35.00 | 8.00 | 0.00 | 3.50 | 3.00 | 6.20 | 25.00 | 72.00 | 2331.86 |

Table S5 Test of significance of Plackett-Burman design

| **Source** | **Sum of Squares** | **df** | **Mean Square** | **F Value** | | **p-value** | **Significance** |
| --- | --- | --- | --- | --- | --- | --- | --- |
| **Model** | 2.568E+005 | 8 | 32105.86 | 4.59 | 0.1186 | | * |
| **A-** Soakig temperature | 48152.34 | 1 | 48152.34 | 6.89 | 0.0081 | | *** |
| **B-** Soaking time | 1014.39 | 1 | 1014.39 | 0.15 | 0.7286 | | * |
| **C- CaCl_2_** | 40.74 | 1 | 40.74 | 5.828E-003 | 0.9440 | | * |
| **D- MSG** | 45066.54 | 1 | 45066.54 | 6.45 | 0.0096 | | *** |
| **E- VB_6_** | 6254.96 | 1 | 6254.96 | 0.89 | 0.4140 | | * |
| **F- pH** | 2040.76 | 1 | 2040.76 | 0.29 | 0.6265 | | * |
| **G-** Germination **t**emperature | 1223.11 | 1 | 1223.11 | 0.17 | 0.7039 | | * |
| **H-Germination time** | 1.531E+005 | 1 | 1.531E+005 | 21.90 | 0.0003 | | *** |
| Residual | 20969.84 | 3 | 6989.95 |  |  | |  |
| Cor total | 2.778E+005 | 11 |  |  |  | |  |
| **R^2^** | 0.9245 |  |  |  |  | |  |
| **Radj^2^**  **Rpred^2^** | 0.7232  6.939 |  |  |  |  | |  |

*No significant different(P＞0.05),**Significant different(P＜0.05＝,***Extremely significant different(P＜0.01＝

Table S6 Test of significance for regression equation coefficients of CCD

| Source | Sum of Squares | df | Mean Square | F Value | p-value | Significance |
| --- | --- | --- | --- | --- | --- | --- |
| Model | 2.42E+005 | 9 | 26892.14 | 5.72 | 0.0058 | *** |
| A_1_-Soaking Temperature | 16207.64 | 1 | 16207.64 | 3.45 | 0.0929 | * |
| B_1_-MSG Concentration | 75336.83 | 1 | 75336.83 | 16.04 | 0.0329 | ** |
| C_1_-Germination Time | 28735.06 | 1 | 28735.06 | 6.12 | 0.0025 | *** |
| A_1_B_1_ | 3339.90 | 1 | 3339.90 | 0.71 | 0.4188 | * |
| A_1_C_1_ | 11398.99 | 1 | 11398.99 | 2.43 | 0.1503 | * |
| B_1_C_1_ | 39264.43 | 1 | 39264.43 | 8.36 | 0.0161 | ** |
| A_1_^2^ | 7455.19 | 1 | 7455.19 | 1.59 | 0.2363 | * |
| B_1_^2^ | 3428.12 | 1 | 3428.12 | 0.73 | 0.4129 | * |
| C_1_^2^ | 55921.95 | 1 | 55921.95 | 11.91 | 0.0062 | *** |
| Residual | 46973.43 | 10 | 10 |  |  |  |
| Lack of fit | 25862.79 | 5 | 5172.56 | 1.23 | 0.4146 | not significant |
| Pure error | 21110.64 | 5 | 4222.13 |  |  |  |
| Cor total | 2.89E+005 | 19 |  |  |  |  |
| R_1_^2^ | 0.8375 |  |  |  |  |  |
| Radj^2^ | 0.6912 |  |  |  |  |  |
| Rpred^2^ | 0.1570 |  |  |  |  |  |

*No significant different(P＞0.05),**Significant different(P＜0.05＝,***Extremely significant different(P＜0.01＝

Table S7 Test of significance for regression equation coefficients of BBD

| Source | Sum of Squares | df | Mean Square | F Value | p-value | Significance |
| --- | --- | --- | --- | --- | --- | --- |
| Model | 1.737E+006 | 14 | 1.241E+005 | 12.03 | < 0.0001 | *** |
| A_2_- Solid-liquid Ratio | 1.810E+005 | 1 | 1.810E+005 | 17.55 | 0.0009 | *** |
| B_2_- Ultrasonic Power | 2351.72 | 1 | 2351.72 | 0.23 | 0.6403 | * |
| C_2_- Ultrasonic Time | 4032.97 | 1 | 4032.97 | 0.39 | 0.5418 | * |
| D_2_- Ultrasonic Temperature | 50980.02 | 1 | 50980.02 | 4.94 | 0.0432 | ** |
| A_2_B_2_ | 25729.76 | 1 | 25729.76 | 2.50 | 0.1365 | * |
| A_2_C_2_ | 32166.42 | 1 | 32166.42 | 3.12 | 0.0992 | * |
| A_2_D_2_ | 1.041E+005 | 1 | 1.041E+005 | 10.09 | 0.0067 | *** |
| B_2_C_2_ | 93119.57 | 1 | 93119.57 | 9.03 | 0.0095 | *** |
| B_2_D_2_ | 31203.46 | 1 | 31203.46 | 3.03 | 0.1039 | * |
| C_2_D_2_ | 8759.09 | 1 | 8759.09 | 0.85 | 0.3723 | * |
| A_2_^2^ | 1.184E+006 | 1 | 1.184E+006 | 114.84 | < 0.0001 | *** |
| B_2_^2^ | 32491.73 | 1 | 32491.73 | 3.15 | 0.0976 | * |
| C_2_^2^ | 77296.31 | 1 | 77296.31 | 7.50 | 0.0160 | ** |
| D_2_^2^ | 10949.26 | 1 | 10949.26 | 1.06 | 0.3203 | * |
| Residual | 1.444E+005 | 14 | 10312.33 |  |  |  |
| Lack of fit | 1.242E+005 | 10 | 12417.48 | 2.46 | 0.2000 | not significant |
| Pure error | 20197.85 | 4 | 5049.46 |  |  |  |
| Cor total | 1.881E+006 | 28 |  |  |  |  |
| R_2_^2^ | 0.9233 |  |  |  |  |  |
| Radj^2^ | 0.8465 |  |  |  |  |  |
| Rpred^2^ | 0.6031 |  |  |  |  |  |

*No significant different(P＞0.05),**Significant different(P＜0.05＝,***Extremely significant different(P＜0.01
